# Epidemic Measles

**Published:** 1900-06-09

**Authors:** 


### The Hospital

A Journal of

The Medical Sciences and Hospital Administration.

Vol. XXVIII.—No. 715.] Edited by Sir Henry Burdett, K.C.B., and Solomon C. Smith, M.D., M.R.C.P. [June 9, 1900.

### Epidemic Measles.

The recently-issued report for 1898-99 of the Medical Officer of the Local Government Board, the last to which the name of the lamented author, the late Sir Richard Thorne-Thorne, will be attached, contains, among a great deal of highly interesting and instructive matter, a history of an epidemic of measles at Burton-upon-Trent which ought to attract the notice alike of sanitary reformers, of school managers, and of parents. The outbreak which gave occasion to the report, and which was investigated by Dr. Theodore Thomson, commenced in January, 1898, and continued until the middle of the following August, or a period of 30 weeks, during which no less than 2,015 cases of the disease were notified to the local authority. The mortality was exceedingly small, amounting only to 29 cases, all but one in children under five years of age, and by far the largest proportion, as is usual, in children between one year old and three. So far, it would seem that local experience supported the too common view that measles is a malady of only moderate severity, attended with but little danger if the patients are properly cared for, and hence scarcely justifying the imposition of burdensome restrictions upon an infected community. Sir Richard Thorne-Thorne, however, calls attention to the fact that the lives of close upon 13,000 children are annually sacrificed to measles in England and Wales, and that, coincidently with the increasing aggregation of children in elementary schools, the tendency of this mortality is to increase rather than to diminish. He, therefore, regards the question of the control of measles as one which calls for sustained and energetic action, not only on the part of the authorities who are concerned with the maintenance of public health, but also, and in systematic co-operation with these, of those who are charged with the education of young children. There is much reason to believe that the diffusion of an epidemic of measles might be checked in some cases if it were to receive due attention from the very first; and that it is mainly the carelessness, alike private and official, which springs from an imperfect perception of the true character and possible severity of the malady, that is answerable for its being so soon and so often permitted to get out of hand, and to assume dimensions with which the ordinary resources of a medical officer of health and his staff are no longer adequate to cope.

Four years have elapsed since Sir Richard Thorne-Thorne last directed attention to this question, then as now on the basis of Dr. Theodore Thomson's inquiries into the circumstances of particular outbreaks, and added the weight of his authority to that gentleman's conclusions and recommendations. Dr. Thomson classified the sources from which local sanitary authorities might derive knowledge of the prevalence and extent of measles in their districts under the three heads of notification, compulsory or voluntary, information from school authorities, and other information, such as might be obtained by virtue of arrangements with Poor Law medical officers, relieving officers, guardians, local registrars, district visitors, and the clergy, and he dwelt strongly upon the importance of utilising all these sources as far as possible in order that each of them might supplement the deficiencies of the rest. In view also of the extreme and early infectiousness of the malady, he regarded schools, from their influence in bringing large numbers of children together, as liable to act as centres of rapid and extensive diffusion, and urged that the managers should be systematically informed in what families and houses the disease had appeared, and advised strictly to exclude from school the apparently healthy members of such families, or the inmates of such houses, until all risk of infection had disappeared. Such a course should not be limited to the public or Board schools, but should extend to private and Sunday schools in the invaded neighbourhood, and its efficient adoption would in many instances render complete closure of schools unnecessary. Such closure, Dr. Thomson thinks, should not be resorted to while there is any reasonable prospect of effectively controlling measles by other means; nor, on the other hand, should it be delayed until any prospect cf benefit from it has well-nigh disappeared. Among other measures recommended are the control of public libraries; the supervision of workers proceeding from infected homes to various industries; and instruction of the public for the purpose of arousing a proper appreciation of the death-dealing effects of measles on the infantile population. Unless proceedings such as these are had recourse to, the ordinary history of an epidemic will be that it will soon pass beyond the control of the sanitary staff of the locality, and that nothing will remain but to gaze helplessly at its continued progress until the susceptible material is exhausted. This seems to have been the course of events at Burton; and Sir Richard Thorne-Thorne asserts that the records of his office do not point to any instance in which the practical measures of precaution have been so combined and enforced that the maximum of good has been attained by their application.

